# Pathogen-Mediated Stomatal Opening: A Previously Overlooked Pathogenicity Strategy in the Oomycete Pathogen *Phytophthora infestans*

**DOI:** 10.3389/fpls.2021.668797

**Published:** 2021-07-12

**Authors:** Li-Na Yang, Hao Liu, Yan-Ping Wang, Jenifer Seematti, Laura J. Grenville-Briggs, Zonghua Wang, Jiasui Zhan

**Affiliations:** ^1^Institute of Oceanography, Minjiang University, Fuzhou, China; ^2^Institute of Plant Virology, Fujian Agriculture and Forestry University, Fuzhou, China; ^3^Department of Plant Protection Biology, Swedish University of Agricultural Sciences, Alnarp, Sweden; ^4^Department of Forest Mycology and Plant Pathology, Swedish University of Agricultural Sciences, Uppsala, Sweden

**Keywords:** stomatal immunity, starch degradation, triacylglycerol breakdown, phytophthora infestans, potato defences

## Abstract

*Phytophthora infestans*, the most damaging oomycete pathogen of potato, is specialized to grow sporangiophore through opened stomata for secondary inoculum production. However, it is still unclear which metabolic pathways in potato are manipulated by *P. infestans* in the guard cell–pathogen interactions to open the stomata. Here microscopic observations and cell biology were used to investigate antagonistic interactions between guard cells and the oomycete pathogen. We observed that the antagonistic interactions started at the very beginning of infection. Stomatal movement is an important part of the immune response of potato to *P. infestans* infection and this occurs through guard cell death and stomatal closure. We observed that *P. infestans* appeared to manipulate metabolic processes in guard cells, such as triacylglycerol (TAG) breakdown, starch degradation, H_2_O_2_ scavenging, and NO catabolism, which are involved in stomatal movement, to evade these stomatal defense responses. The signal transduction pathway of *P. infestans*-induced stomatal opening likely starts from H_2_O_2_ and NO scavenging, along with TAG breakdown while the subsequent starch degradation reinforces the opening process by strengthening guard cell turgor and opening the stomata to their maximum aperture. These results suggest that stomata are a barrier stopping *P. infestans* from completing its life cycle, but this host defense system can be bypassed through the manipulation of diverse metabolic pathways that may be induced by *P. infestans* effector proteins.

## Introduction

Stomata, bordered by a pair of guard cells, play several essential roles in many biological and biochemical processes of terrestrial plants. The size of a stomatal aperture is dynamically regulated by the integration of environmental signals and endogenous hormonal stimuli. Under light stimulation and/or high humidity, stomata open to promote carbon dioxide and oxygen flow for photosynthesis and water evaporation (Berry et al., 2010; Buckley, 2019) and *vice versa*. Biotic stressors, such as pathogens, also regulate the stomatal aperture of plants. It has long been noticed that many prokaryotic plant pathogens use stomata as a gate to penetrate the inner tissue of plants. Some pathogens, for example, the bacterium *Xanthomonas campestris* pv *armoraciae* (Hugouvieux et al., 1998), fungi from the *Puccinia* Genus (Shafiei et al., 2007), and the oomycete *Plasmopara viticola* (Allègre et al., 2007) are specialized to penetrate and colonize plant tissues only through stomatal pores. For other pathogens, such as the oomycete *P. infestans*, stomata are not essential for invasion and colonization but are required for sporulation (Farrell et al., 1969). To prevent pathogen ingress and reproduction, plants have evolved mechanisms to close stomata upon a perception of pathogens, but adapted pathogens can trigger stomatal reopening to overcome this layer of defense by releasing pathogenicity compounds, such as phytotoxins or effector proteins (Melotto et al., 2017; Ye et al., 2020).

Many compounds, including starch, lipids, and oxidative radicals such as hydrogen peroxide (H_2_O_2_), are involved in signal transduction to control stomatal movement (Shimazaki et al., 2007; Horrer et al., 2016; McLachlan et al., 2016) and the same compounds may also be manipulated by pathogens to overcome stomata-mediated defenses. Starch, synthesized in plastids in both photosynthetic and non-photosynthetic cells, is the principal carbohydrate storage of higher plants (Zeeman et al., 2010). In guard cells, starch degradation provides organic acids and sugars to increase guard cell turgor pressure and promote the stomatal opening. For example, glucose derived from starch degradation was found to be responsible for rapid stomatal opening in Arabidopsis after exposure to blue light (Flütsch et al., 2020). Furthermore, malate is recognized unequivocally as the predominant donor of the organic anions needed to balance the positive charge of K^+^ ions during stomatal opening (Santelia and Lawson, 2016).

Triacylglycerol (TAG), a dominant lipid compound for energy storage present as lipid droplets (LDs) in guard cells, is also involved in the stomatal opening that is stimulated by light illumination (McLachlan et al., 2016). The abundance of TAG in the guard cells is significantly reduced in response to light, and PHOT blue light receptors are involved in this response (McLachlan et al., 2016). Stomatal movement is also an energy-demanding process. The abundant TAGs in guard cells ensure the generation of adequate ATP and activation of the proton pumps required for stomatal opening, such as plasma membrane H^+^-ATPases (McLachlan et al., 2016).

The reactive oxygen species hydrogen peroxide (H_2_O_2_) and the reactive nitrogen species nitric oxide (NO) have a wide range of effects on the developmental processes and stress responses of plants, including seed germination, root development, drought resistance, and defense against pathogens (Liao et al., 2012; He et al., 2013; Smirnoff and Arnaud, 2019). The concurring dynamics of H_2_O_2_ and NO in most plant organs suggest that they are likely metabolized in parallel and act in tandem (Tanou et al., 2009; Clark et al., 2010), although some results show that NO may function downstream of H_2_O_2_ (Zhang et al., 2017). In guard cells, H_2_O_2_ and NO are key regulators that work synergistically or independently in regulating stomatal movement. In the last few years, the roles and mechanisms of ABA-induced stomatal closure modulated by H_2_O_2_ and NO have been exploited widely (Jannat et al., 2011; Rodrigues et al., 2017). Additionally, H_2_O_2_ and NO are also involved in darkness-induced stomatal closure (Desikan et al., 2004; Zhang et al., 2017). H_2_O_2_ and NO concentrations in guard cells increase in darkness but decrease in light.

*Phytophthora infestans*, the causal agent of potato (*Solanum tuberosum* L.) late blight disease, which was responsible for the Irish potato famine in the 1840s, is to date one of the most devastating plant pathogens known to man (Fry, 2008). *P. infestans* continues to be a major yield-limiting factor in potato production. However, it is also a model species to study the biology, genetics, and evolution of host–pathogen interactions in the oomycetes (Wang et al., 2017; Yang et al., 2019). Despite the existence of sexual reproduction, *P. infestans* still reproduces primarily in an asexual manner by forming sporangia (Zhu et al., 2015). Sporogenesis is an important part of the asexual cycle and massive numbers of sporangia (up to 300,000 per lesion) can be produced rapidly and dispersed across whole fields within days (Fry, 2008). In this process, potato stomata acts as the physical barrier that *P. infestans* must break through, to allow sporangia to be released from the plant, and *P. infestans* sporangiophores are specialized to grow out through stomatal apertures (Farrell et al., 1969).

Recently, a non-specific lipid transfer protein, *StLTP10*, was found to regulate stomatal closure in potato after *P. infestans* infection by physical interaction with the ABA receptor PYL4, indicating that stomatal immunity is important in potato defense against *P. infestans* (Wang et al., 2020). In the current study, cellular interactions between the potato guard cells and *P. infestans* were explored. We found that the antagonistic interactions between the potato guard cells and *P. infestans* started at the very beginning of infection. The non–race-specific stomatal closure caused by guard cell death was found in more than 10 potato cultivars varying in genetic background and quantitative resistance, indicating that guard cell suicide is deployed as a common immune response of potato against *P. infestans* infection. However, we further showed that this immune response is suppressed by the pathogen through the regulation of starch, TAG, H_2_O_2_, and NO metabolism. We hypothesized that these biochemical processes may be induced by pathogen-effector proteins. The signal transduction pathway of the pathogen-induced stomatal opening may start from H_2_O_2_ and NO scavenging and TAG breakdown, proceed through starch degradation, and end up with a stomatal aperture maximized for the growth of sporangiophores upward through the stomata, for aerial release of sporangia.

## Materials and Methods

### Growth and Maintenance of Potato and *Phytophthora infestans*

Potato cv Desiree plants were grown at 19°C and 60% humidity in a greenhouse supplemented with 16 light-hours at the intensity of 120–150 μmol/m/^2^/s. Leaves used for experiments were taken from 5- to 6-week-old plants. *P. infestans* isolate A21b collected from Fuqing, Fujian Province, in 2016, with high sporangia yield and isolate 88069 (A1 mating type, race 1.3.4.7) retrieved from long-term storage, were grown on Rye B plates at 18°C in dark. After two weeks, the plates were flooded with 5 ml of sterilized water and scraped with a plastic rod to make a sporangial suspension. The suspension was calibrated to ∼80,000 sporangia/ml using a hemocytometer and was sprayed evenly on the abaxial side of potato leaves to ensure sufficient and uniform infection. After the inoculation, the potato leaves were kept in sealed boxes to maintain moisture and they were placed at 18°C in dark. The inoculation was replicated at least three times for each treatment.

### Apoplastic Fluid Collection and Infiltration

Apoplastic fluids (AFs) were collected from potato leaves infected with *P. infestans* A21b when disease symptoms were visible but the detached leaves were still green, which usually occurred around 20 h post inoculation (hpi). After removal of sporangia and mycelia, the infected leaves were immersed in distilled water in a beaker and then infiltrated by placing them into a vacuum desiccator for 5 min. Excess water droplets on leaf surfaces were removed with tissue paper. The infiltrated potato leaves were rolled up and inserted into 30 ml tubes with small holes at the bottom. Each tube was then slipped into a larger, 50 ml tube and centrifuged at 1,000 × *g* for 10 min at 10°C. The harvested AF from the larger tubes was filtered through 0.22 μm Millex sterile filters and used to infiltrate potato leaves. Three leaves per potato plant were infiltrated with the AF and three plants were included for each treatment, bringing nine leaves in total for each treatment. AF collected from uninfected healthy potato leaves was used as a control. Diphenyl methylphosphonate (DMP), when used, was co-infiltrated with AFs at a concentration of 25 μM (from a 25 mM stock in DMSO).

### Stomatal Aperture Measurements

Unless stated otherwise, all experiments were conducted in dark and the potato leaves used in the study were kept in the dark at 18°C for 2 h before use to ensure stomata closure. During the infection time course, the detached leaves with the abaxial side up were attached to glass slides using double-sided adhesive tape and photographed at 20X magnification using a microscope (NIKON Ni-U). Epidermal strips were manually peeled off from the infection sites at 12 and 24 hpi, and incubated in KCl/MES buffer (10 mM MES, 5 mM KCl, and 50 μM CaCl_2_) with ABA, CaCl_2_, H_2_O_2_, Sodium Nitroprusside (SNP), and Na_3_VO_4_ at 18°C in dark. After 2 h of incubation, the epidermis was photographed using a microscope (NIKON Ni-U) at 20X magnification. Stomatal apertures were measured using the software ImageJ 1.50. All the experiments were repeated in at least three independent treatments, and three leaves from different plants were used per treatment.

### Starch Quantification in Guard Cells

Starch in guard cells was quantified by the pseudo-Schiff propidium iodide (PS–PI) staining protocol as described previously (Horrer et al., 2016) with some minor modifications. Briefly, the epidermis was manually peeled off the detached potato leaves that had either been infected with *P. infestans*, infiltrated with AF, or treated with light incubation and fixed in 50% (v/v) methanol, 10% (v/v) acetic acid at 4°C overnight. The epidermal peels were rinsed briefly with sterilized water and incubated in 1% periodic acid at room temperature for 40 min. They were rinsed again with sterilized water and stained with Schiff reagent (100 mM sodium metabisulfite and 0.15 N HCl) and propidium iodide [0.1 mg/ml (w/v) final concentration] for 1–2 h. The stained epidermal peels were affixed to microscope slides and submerged in chloral hydrate solution overnight. Excess chloral hydrate on the epidermal peels was removed from the microscope slides and the epidermal peels were fixed with Hoyer’s solution. The images of fluorescent activity in the guard cells were taken using a NiKON (Ni–U) fluorescence microscope with an excitation wavelength of 540 nm and an emission wavelength of 605 nm. The starch granule area was quantified by measuring fluorescent areas in the guard cells with ImageJ 1.50. This experiment was repeated using three independent treatments, and three leaves from different plants were used in each treatment.

### Lipid Droplet Quantification in Guard Cells

After *P. infestans* infection, AF infiltration or light treatment, the leaf epidermis was manually peeled from the detached leaves, at the specified time points described above, and incubated in 30 μM Nile Red (NR; McLachlan et al., 2016) for 40 min and washed in KCl/MES buffer (10 mM MES, 5 mM KCl, and 50 μM CaCl_2_) for 5 min. Images of NR fluorescence activity in the guard cells were taken using a NiKON (Ni–U) fluorescence microscope with excitation wavelengths of 465–495 nm and emission wavelengths of 512–558 nm and the LD volume was quantified using ImageJ 1.50. This experiment was repeated using three independent treatments, and with three leaves from different plants in each treatment.

### H_2_O_2_ and NO Accumulation Measurement

After *P. infestans* inoculation, the epidermis was manually peeled off from the detached leaves at each specified time point and incubated for 20 min in KCl/MES buffer with 50 μM H_2_DCF DA for H_2_O_2_ measurements or in KCL/MES buffer with 10 μM DAF-FM DA for NO measurements and then washed with KCl/MES buffer twice. The images of H_2_O_2_ and NO fluorescent activity in the guard cells were taken using a Nikon fluorescence microscope (Ni–U) with excitation wavelengths of 465–495 nm and emission wavelengths of 512–558. H_2_O_2_ and NO concentrations were quantified by measuring their fluorescence density in the guard cells using ImageJ 1.50. Again, this experiment was repeated using three independent treatments, and with three leaves in each treatment.

The accumulation of H_2_O_2_ in mesophyll cells was measured by histochemical analysis *via* 3,3′-diaminobenzidine (DAB) staining. Potato leaves which were inoculated with or without (control, CK) *P. infestans* sporangia of isolate A21b were incubated in DAB solution (1 mg/ml, pH 3.8) for 16 h at 25°C in the dark, then soaked in 95% ethanol overnight to remove chlorophyll (Thordal-Christensen et al., 1997). Photos were taken using a digital camera.

### RNA Extraction

RNA was extracted as described previously (Resjö et al., 2017). Briefly, the total RNA was extracted from frozen samples ground in liquid nitrogen using a Qiagen RNeasy Plant Mini kit following the protocol set by the manufacturer. Samples were derived from a time-course of potato leaves (cultivar Desiree) inoculated with *P. infestans* strain 88069, or from pre-infection structures collected *in vitro* as described (Grenville-Briggs et al., 2008). Before cDNA synthesis all samples were DNase treated using the Ambion Turbo DNA-free kit, according to the protocol set by the manufacturer. RNA samples were assessed for purity and integrity by agarose gel electrophoresis and Nanodrop Spectrophotometry. First strand cDNA was synthesized from 20 g total RNA by oligo(dT) priming using the Superscript IV Reverse transcriptase cDNA synthesis kit (Thermo Scientific).

### Quantitative RT-PCR Assays

The primer pairs that annealed specifically to each of the candidate effectors PITG_11755 (Meijer et al., 2014) and PITG_15152 (de Vries et al., 2017) were used to quantify gene expression *in vitro* and *in planta* as described previously (Resjö et al., 2017). A template cDNA for *in planta* analysis over an infection time course was derived from mycelium grown for 72 h in liquid pea broth as well as from potato leaves inoculated with *P. infestans*. Samples were taken at 6, 12, 24, and 48 hpi. Pre-infection samples of non-sporulating mycelium, sporangia, zoospores, germinating cysts, and germinating cysts with appressoria were collected as described by Grenville-Briggs et al. (2008). The actA gene from *P. infestans* was used as constitutively expressed endogenous control and the abundance of each transcript in mycelium was determined relative to the actA transcript as described previously (Grenville-Briggs et al., 2008). All qRT-PCR assays were performed using three biological replicates. The results from each assay were analyzed using the modified ΔΔcT method, and relative expression was determined relative to a calibrator sample (mycelium) as described previously (Resjö et al., 2017).

### Plasmid Construction and Transient *in planta* Expression

The full-length sequences of PITG_11755 and PITG_15152 without their signal peptides were cloned from the gDNA of *P. infestans*, ligated into pEarlyGate 101 (C-terminal GFP tag) and pEarlyGate 104 (N-terminal GFP tag), respectively, using Vazyme ClonExpressII One Step Cloning Kit, and then transformed into *Agrobacterium tumefaciens* strain AGL1. Overnight, *A. tumefaciens* cultures were harvested by centrifugation and resuspended in infiltration buffer (10 mM MES, 10 mM MgCl_2_, and 200 mM acetosyringone). The resuspended *A. tumefaciens* cells with an optical density (OD_600_) of 0.5 were infiltrated into leaves of 5- to 6-week-old potato plants. Stomatal apertures were measured 3–5 days post infiltration.

### Statistical Analyses

Analysis of variance (ANOVA) for stomatal aperture and concentrations of starch, lipids, H_2_O_2_, and NO in the guard cells were performed using the general linear model embedded in SAS 9.4, and significant differences between treatments in these parameters were evaluated using a Duncan test. Standard deviation was estimated separately for each parameter using the data generated from different replicates and is shown as error bars in the displayed charts.

## Results

### Starch Degradation and Triacylglycerol Breakdown Are Associated With Light-Induced Stomatal Opening in *Solanum tuberosum*

**FIGURE 1:**
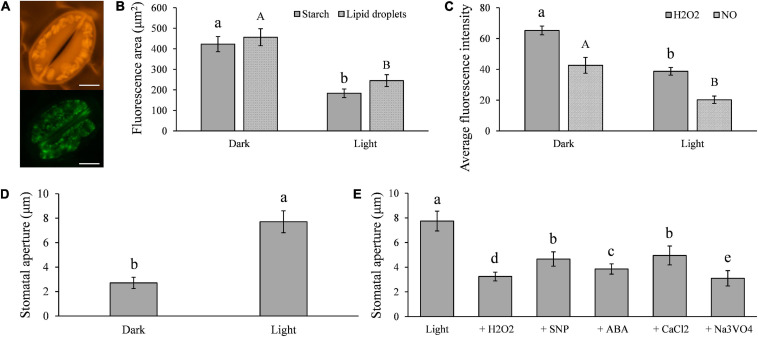
Light-induced stomatal opening in potato is associated with the metabolisms of triacylglycerols (TAGs), starches, H_2_O_2_, NO, ABA, CaCl_2_, and plasma membrane H^+^-ATPase in guard cells: **(A)** images showing potato guard cells containing a large amount of starch (up) and TAGs (below); **(B)** compared with the results seen in dark conditions, the quantities of starch and TAGs in guard cells were significantly less in light (100 μmol/m^2^/s^1^ for 6 h); **(C)** H_2_O_2_ and NO contents in guard cells were significantly decreased in light compared with those in dark conditions; **(D)** the size of stomatal aperture significantly increased in light compared with those in dark conditions; and **(E)** light-induced stomatal opening was impaired by the artificial supplement of 2 μM H_2_O_2_, 1 μM NO, 10 μM ABA, 5 mM CaCl_2_, and 1 mM Na_3_VO_4_. The marker = 20 μm. Photos within a panel are from a representative of a single replicate while photos among panels are pooled from multiple replicates.

It has been documented that LDs (Sakaki et al., 1995) and starch (Pallas, 1964) are present widely in both higher and lower plants, and their catabolism is associated with light-induced stomatal opening (Horrer et al., 2016; McLachlan et al., 2016). To verify these reports in potato (*Solanum tuberosum*), we measured the LD and starch contents in cv Desiree leaves under both dark and light conditions. We found that large amounts of starch and LDs are present in potato guard cells (Figure 1A), and that the contents of the two compounds were significantly decreased under light conditions (Figure 1B) when stomatal apertures increased (Figure 1D). The H_2_O_2_ and NO contents of the guard cells were also reduced under the same conditions (Figure 1C). Artificial supplements of H_2_O_2_, NO, ABA, CaCl_2_, and the H^+^-ATPase inhibitor Na_3_VO_4_, all significantly impaired the light-induced stomatal opening (Figure 1E). These results indicate that starch, TAG, H_2_O_2_, and NO are involved in stomatal opening and closing in potato.

### Stomatal Defense Is Inhibited by *Phytophthora infestans*

**FIGURE 2:**
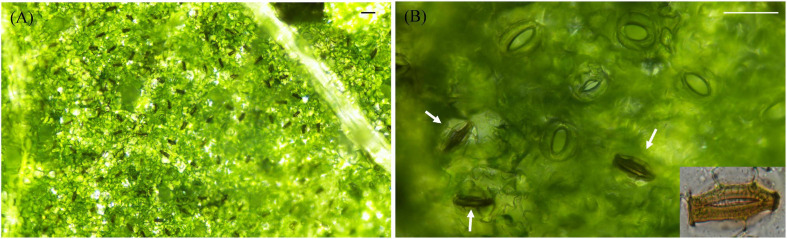
Images showing that potato defenses against *P*hytophthora *infestans* infection at the interface of stomata: **(A)** hypersensitive-like cell death occurred specifically in guard cells 8 h post infection; and **(B)** permanent closure of stomata during the whole infection course after the death of guard cells. The marker = 50 μm. Photos within a panel are from a representative of a single replicate while photos among panels are pooled from multiple replicates.

Many pathogens, particularly prokaryotic microbes, such as bacteria, rely on plant stomata for penetration and infection initiation (Melotto et al., 2006). To prevent the attack, guard cells can perceive bacteria and trigger stomatal closure (Melotto et al., 2017), and even specifically commit suicide, for example, in response to rust fungal invasion (Ye et al., 2020). The appressorium and invading hypha of *P. infestans* can be formed 8–12 hpi (Supplementary Figure 1). The life cycle of the pathogen can be completed within 5 days on potato foliage (Supplementary Figure 2) and the whole field can be transformed from slightly diseased to nearly completely destroyed within ∼2 weeks under ideal conditions (Fry, 2008). Recently, it was demonstrated that stomatal defense may also play a role in potato immunity to *P. infestans* (Wang et al., 2020). Here we monitored the stomata–*P. infestans* interaction over the infection time course under dark conditions to determine whether *P. infestans* can perturb this process. Interestingly, we found a hypersensitive-like response of ∼50% guard cells at the infection sites, where they turned dark brown, atrophied, and eventually died. This process started usually between 4 and 8 h post sporangial inoculation (hpi) in cv. Desirée, leading to the permanent closure of these stomata (Figure 2). No sporangiophores were observed to emerge through the dead stomata. Besides Desiree, the same pattern was found in 18 other potato cultivars with varying resistance levels against *P. infestans* (Supplementary Figure 3). Thus, this phenomenon appears to be a general potato response to *P. infestans* infection and not race-cultivar specific.

**FIGURE 3:**
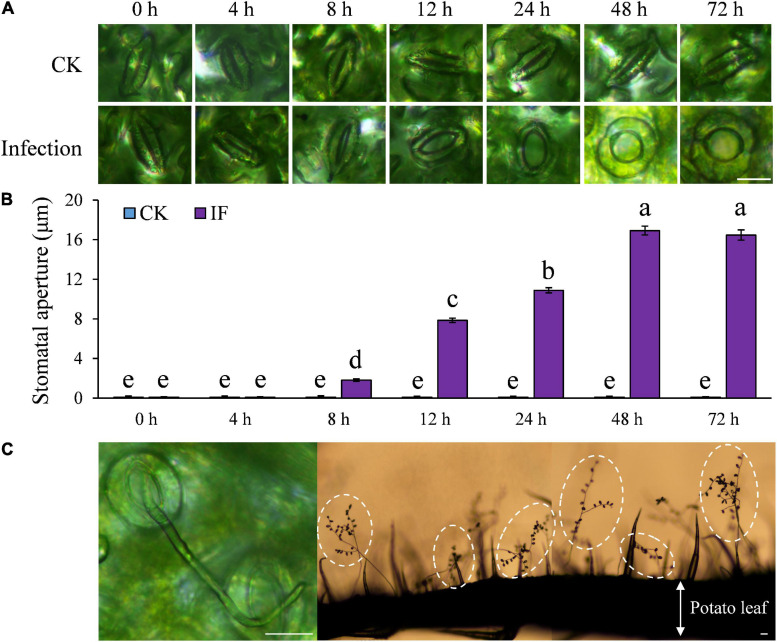
Stomatal aperture was significantly increased after *P. infestans* inoculation under dark condition: **(A)** Images showing stomatal aperture of potato leaves increased after the successful colonization of *P. infestans*; **(B)** stomatal aperture started to increase from 8 h post infection (hpi), and reached the maximum size at 48 hpi; and **(C)** images showing that sporangiophores emerged through opened potato stomata 72 hpi and a large amount of sporangia (the lemon-shaped spots in dashed circles) were formed 4–5 days post inoculation. The marker = 20 μm. Photos within a panel are from a representative of a single replicate while photos among panels are pooled from multiple replicates.

On the other hand, we found the majority of leaf stomata at the infection sites started to open at 8 hpi of *P. infestans*, reached their maximum aperture at 48 hpi and afterward remained fully open (Figures 3A,B). Although initially some guard cell death (∼50%) was observed, no further dead guard cells were found after the pathogen-induced stomatal opening started. Sporangiophores started to emerge from opened stomata at the infection sites around 72 hpi and large amounts of sporangia were observed after 4–5 days post inoculation in cv. Desirée leaves (Figure 3C and Supplementary Figure 4). The infection-induced stomatal opening was also found in 18 other potato cultivars (Supplementary Figure 3). These results suggest that potato plants can sense and defend against *P. infestans* infection by stomatal closure, while *P. infestans* can suppress these defenses in potato as found in other plant–pathogen interactions.

### *Phytophthora infestans* Infection Induces Potato Stomatal Opening by Degrading Starch in Guard Cells

**FIGURE 4:**
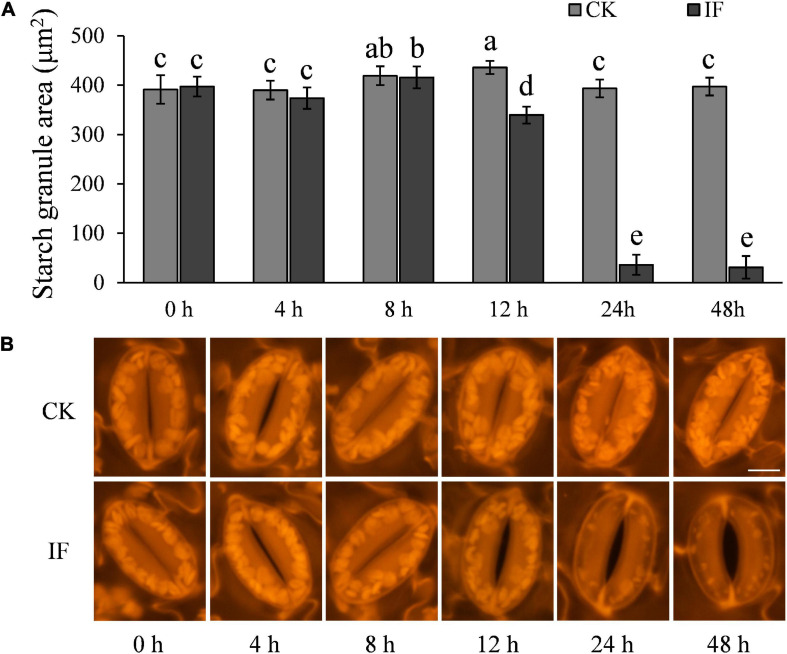
*P. infestans*-induced stomatal opening correlates with starch degradation in guard cells: **(A)** starches in guard cells of *P. infestans*-infected potato leaves started to degrade at 12 hpi in dark, and at 24–48 hpi nearly no starch was visible in the guard cells; **(B)** images show starch in the guard cells of control (CK, no *P. infestans* inoculation) and infected (IF) potato leaves. Starches in the guard cells around the infection sites started to degrade 12 hpi. The marker = 10 μm. Photos within a panel are from a representative of a single replicate while photos among panels are pooled from multiple replicates.

As starch degradation is strongly connected to stomatal opening (Horrer et al., 2016) and potato guard cells contain a large amount of starch (Figure 1A), we hypothesized that *P. infestans* may manipulate potato stomatal movement by inducing starch degradation in the guard cells. To test this hypothesis, we examined starch dynamics in the guard cells during *P. infestans* infection. Indeed, we found that starch metabolic processes in the guard cells were altered by *P. infestans* infection. Starch in guard cells at the infection sites started to degrade at 12 hpi (Figure 4). At 24 and 48 hpi, only remnant starch grains were observed in the guard cells of stomata, indicating that they are almost completely degraded (Figure 4).

**FIGURE 5:**
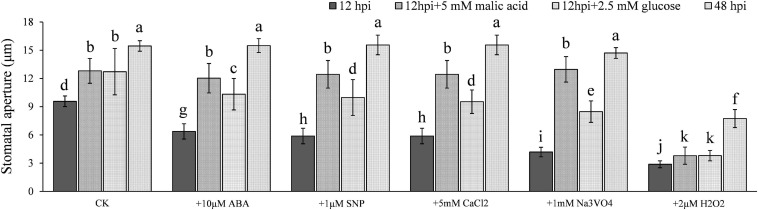
Glucose and malate accumulations were responsible for the full stomatal opening during *P. infestans* infection. Stomatal apertures reached the maximum 48 hpi and failed to the stimulation by ABA, NO, CaCl_2_, and Na_3_VO_4_. Glucose and malate supplement to epidermal strips collected 12 hpi showed a similar trend. Photos within a panel are from a representative of a single replicate while photos among panels are pooled from multiple replicates.

The metabolism of both glucose and malate has previously been reported during stomatal opening (De Angeli et al., 2013; Santelia and Lawson, 2016). This led us to hypothesize that the significant increase of stomatal aperture 12–24 hpi may result from glucose or malate accumulation. To test this, we treated epidermal strips collected at 12 hpi, that is, the time starch started to degrade, with 2.5 mM glucose and 5 mM malate, respectively, and found that glucose and malate supplements indeed facilitated stomatal opening significantly (Figure 5), although the stomatal aperture did not reach the same size as that seen at 48 hpi (at the time starch was almost completely degraded). Interestingly, after starch degraded completely and the apertures reached maximum, which occurred at ∼48 hpi, stomata lost responsiveness to the stimulation of ABA, NO precursor SNP, CaCl_2_, and Na_3_VO_4_. The epidermal strips supplemented with malate at 12 hpi showed the same phenotype (Figure 5). Glucose supplement also weakened the stimulation of ABA, NO precursor SNP, CaCl_2_, and Na_3_VO_4_ (Figure 5). These results indicate that starch degradation was affected by *P. infestans*, which we hypothesized may induce stomatal opening, possibly aided by soluble sugars, such as glucose and malate.

### *Phytophthora infestans* Infection Induces Potato Stomatal Opening by TAG Breakdown in Guard Cells

**FIGURE 6:**
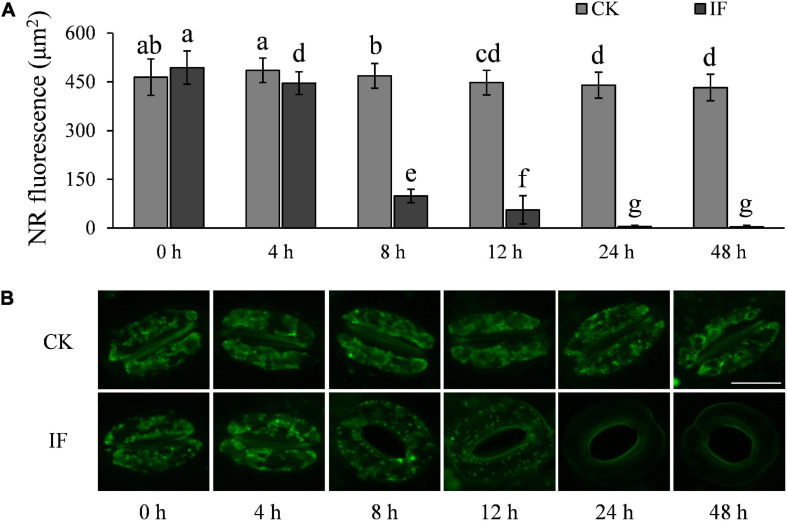
*P. infestans*-induced stomata opening correlates with TAG breakdown in guard cells: **(A)** TAGs in the guard cells of *P. infestans-*infected (IF) potato leaves started to decrease significantly 8 hpi; and **(B)** images showing the volume of TAGs in guard cells of the *P. infestans*-infected (IF) and not-infected (CK) potato leaves. The marker = 20 μm. Photos within a panel are from a representative of a single replicate while photos among panels are pooled from multiple replicates.

One interesting finding is that stomata at the infection sites started to open at 8 hpi but starch degradation in guard cells was not observed until 12 hpi. Therefore, we hypothesized that there must be other metabolic pathways that are also involved in the initiation of the stomatal opening process. It has been shown that TAG in guard cells is the energy source used to activate the proton pump H^+^-ATPase involved in light-induced stomatal opening (McLachlan et al., 2016). We thus monitored TAG dynamics in the guard cells of *P. infestans*-infected and control (CK, not challenged by the pathogen) potato leaves over a 48-h period under dark conditions. We observed that TAG breakdown in the infected leaves started to occur at 8 hpi or earlier (Figure 6), which was almost coincident with the starting time of stomatal opening (Figure 3B). After 24 hpi, TAG was largely absent in the guard cells of the *P. infestans*-infected leaves (Figure 6). These results indicate that TAG breakdown in potato guard cells occurs earlier than starch degradation during the response to *P. infestans* infection.

### H_2_O_2_ and NO Accumulation in Potato Guard Cells Is Disturbed by *Phytophthora infestans* Infection

**FIGURE 7:**
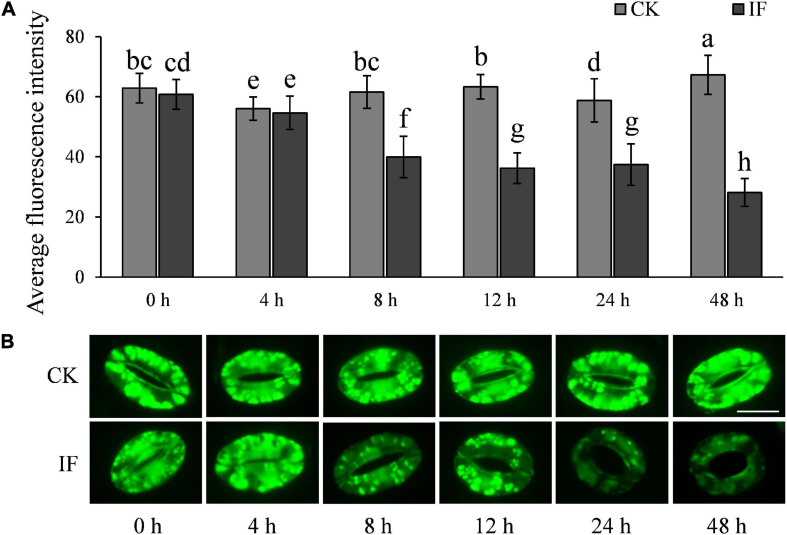
H_2_O_2_ accumulation changes in guard cells during *P. infestans* infection: **(A)** H_2_O_2_ dynamics in the *P. infestans*-infected (IF) and non-infected (CK) potato guard cells; and **(B)** images showing the fluorescence intensity of H2-DCFDA in IF and CK potato guard cells. The marker = 20 μm. Photos within a panel are from a representative of a single replicate while photos among panels are pooled from multiple replicates.

**FIGURE 8:**
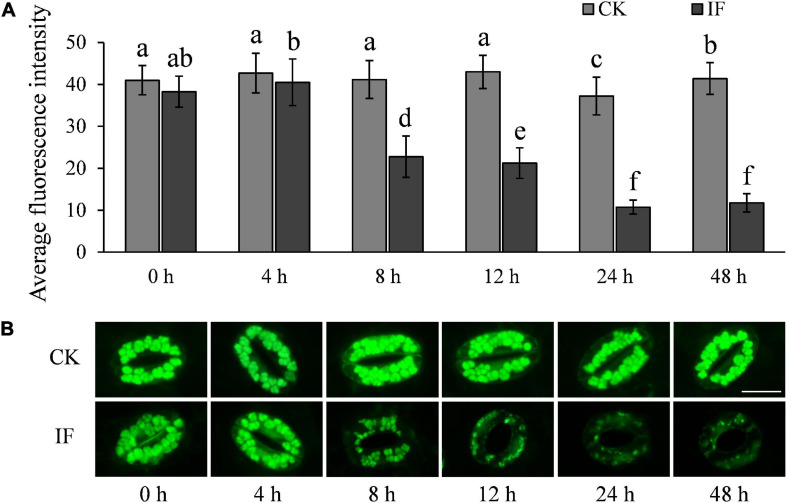
NO accumulation changes in potato guard cells during *P. infestans* infection: **(A)** NO dynamics in the *P. infestans*-infected (IF) and non-infected (CK) potato guard cells; and **(B)** images showing the fluorescence intensity of DAF-FM DA in IF and CK potato guard cells. The marker = 20 μm. Photos within a panel are from a representative of a single replicate while photos among panels are pooled from multiple replicates.

H_2_O_2_ and NO are important signaling molecules involved in stomatal movement, especially in ABA- and dark-induced stomatal activity (Desikan et al., 2004; Jannat et al., 2011; Rodrigues et al., 2017; Zhang et al., 2017). H_2_O_2_ and NO are accumulated in guard cells in response to ABA enrichment and dark stimulation, but are reduced when stomata open in light (Desikan et al., 2004; She et al., 2004). To investigate whether *P. infestans*-induced stomatal opening is associated with H_2_O_2_ and NO metabolism, we measured the two compounds in potato guard cells using the fluorescent dyes dichlorodihydrofluorescein diacetate (H_2_-DCFDA) and 3-amino, 4-aminomethyl-2′, 7′-difluorescein, diacetate (DAF-FM DA), respectively. In contrast to mesophyll cells (Supplementary Figure 5), H_2_O_2_ and NO were found to be significantly reduced in guard cells at the time stomata started to open, that is, 8 hpi (Figures 7, 8). After 8 hpi, NO levels in the infected guard cells continued to fall but H_2_O_2_ levels basically flattened out (Figures 7, 8). These results suggest that the metabolism of H_2_O_2_ in the guard cells is independent from that of the mesophyll cells, and a reduction of both H_2_O_2_ and NO in the guard cells may participate in the *P. infestans*-induced stomatal opening process in potato plants.

### Apoplastic Fluids and Effector Overexpression Induce Stomatal Opening

**FIGURE 9:**
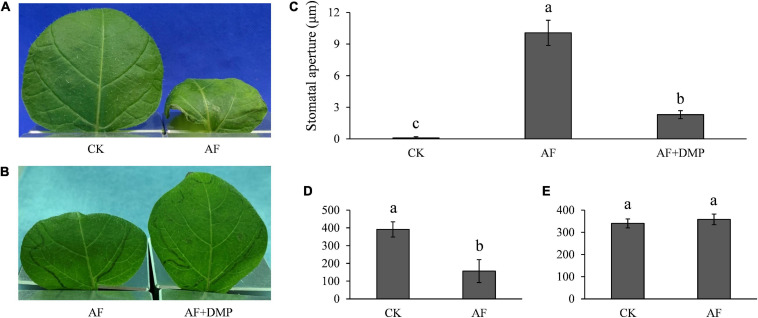
Apoplastic fluids (AFs) induced stomatal opening and TAG breakdown: **(A)** accelerated water evaporation in AF infiltrated potato leaves; **(B)** accelerated water evaporation by AF infiltration was reversed by DMP; **(C)** stomata was opened by AF treatment and the aperture size was compromised by DMP; **(D)** reduced TAGs in potato guard cells by AF treatment; and **(E)** AF had no effect on starch content at 15 h post inoculation in potato guard cells. Photos within a panel are from a representative of a single replicate while photos among panels are pooled from multiple replicates.

During infection, *P. infestans* secretes a large number of apoplastic and cytoplasmic effector proteins which are targeted to the cytoplasm or apoplast of host cells (Haas et al., 2009). To check whether infection-derived molecules, such as effector proteins participate in the antagonistic interactions between potato guard cells and *P. infestans*, AFs that contain a mixture of *P. infestans* secreted effectors were collected from diseased plants and infiltrated into healthy potato leaves. We found that the stomatal aperture was significantly increased, and TAG in the potato guard cells was significantly decreased at 15 h after the infiltration (Figures 9A,C,D). Because AF extraction is known to often damage some of the plant cells, and plant materials leaking from the cytoplasm may complicate the results, we controlled for this by comparatively infiltrating AFs collected from control (uninoculated) plants. The results showed that stomatal apertures and TAG levels were not disrupted by the control AF infiltrations. When we co-infiltrated the AFs from diseased plants with DMP, the LD mobilization inhibitor that acts early in the β-oxidation pathway, the stomatal opening was significantly inhibited (Figures 9B,C). We also found that the content of both H_2_O_2_ and NO decreased in most of the guard cells infiltrated with AF from disease plants (Supplementary Figure 6). However, starch content in the guard cells did not change at 15 h after these AF infiltrations (Figure 9E), and only decreased in some of the cells at 24 h after infiltration when leaves withered (Supplementary Figure 7). These results indicate that infection AFs contain metabolites or proteins, such as effectors of the pathogen, that either directly or indirectly (e.g., general suppression of immune responses) trigger stomatal opening.

**FIGURE 10:**
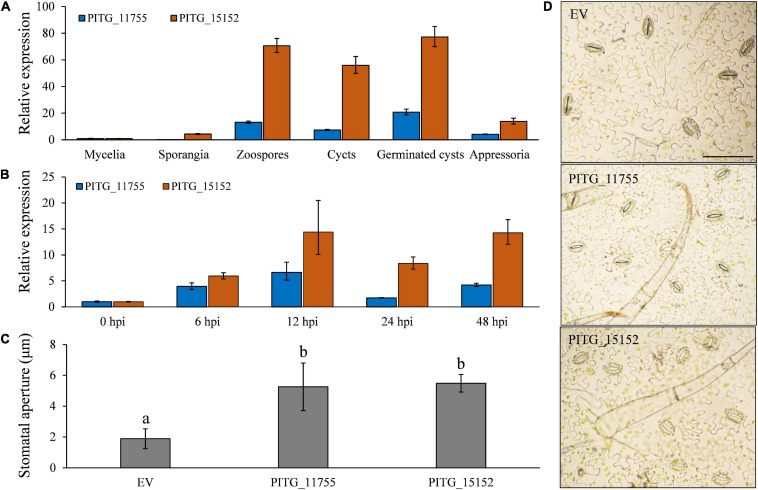
Both extracellular and intracellular effector proteins participate in *P. infestans* infection-induced stomata opening. **(A)** The putative effectors PITG_11755 and PITG_15152 were highly expressed during pre-infection development and **(B)** at both early and late time points *in planta*; **(C)** the overexpression of PITG_11755 and PITG_15152 significantly increased the stomatal aperture; and **(D)** images show increased stomatal apertures after effector overexpression. Photos within a panel are from a representative of a single replicate while photos among panels are pooled from multiple replicates.

The apoplastic candidate effector PITG_11755 and cytoplasmic candidate effector PITG_15152 were both highly expressed in the pre-infection stages and during infection (Figures 10A,B). During a time course of infection, both of these effector genes followed a similar expression pattern, which is highly elevated early on in infection at 6 hpi, rising to a peak at 12 hpi and reduced at 24 hpi before rising again at 48 hpi (Figure 10B). These results suggest that these effectors may have roles both early (at a similar time point to our observations of the onset of stomatal opening) and later on in infection (when sporangiophores are produced and begin to grow out of stomatal openings). To test our hypothesis that effector proteins may be involved in the infection-induced stomatal opening, PITG_11755 and PITG_15152 were non-endogenously overexpressed in potato leaves. We found that both the effector proteins significantly increased stomatal opening at 3 days after infiltration (Figures 10C,D), supporting our hypothesis that effectors of both apoplastic and cytoplasmic origin may have a role, directly or indirectly, in regulating the stomatal opening of potato after *P. infestans* infection.

## Discussion

Stomata are the battle field of molecular and physical interactions between plants and pathogens. On the one hand, many plant pathogens, in particular bacterial pathogens, rely on plant stomata as the natural gate of invasion and colonization (Melotto et al., 2006). On the other hand, since stomata are an inseparable part of the integral innate immune system (Melotto et al., 2017), plants can sense the chemical and physical presence of pathogens and force the closure of stomata to prevent pathogen entrance. This stomatal closure can be achieved in a very short time (< 1 h) through the expression of pattern recognition receptors in guard cells (Robatzek et al., 2006; Liu et al., 2009), such as FLS2, EFR, and CERK1 which can recognize flg22, elf18, elf26, lipopolysaccharide, and chitin of bacterial pathogens (Murata et al., 2015). Recent studies have documented that highly adapted pathogens can produce phytotoxins, such as COR (Bender et al., 1999), or secrete effectors (Jiang et al., 2013; Hurley et al., 2014; Lozano-Durán et al., 2014; Zhou et al., 2015; Wang et al., 2016) to circumvent the host defense system through stomatal closures.

The available knowledge on pathogen-mediated stomatal movements and defenses are exclusively derived from systems involving prokaryotic pathogens and fungi. It was found that plants can defend against bacteria and rust fungi through stomatal closure or guard cell death (Melotto et al., 2006, 2017; Ye et al., 2020). *P. infestans* can penetrate potato epidermal cells directly through the formation of appressoria, therefore stomata are not essential for the penetration and colonization of the pathogen (Farrell et al., 1969,Grenville-Briggs et al., 2008). However, the stomatal opening is required for the discharge of sporangia, the main secondary inoculum source leading to late blight epidemics. In this study, we demonstrate that stomatal movement and defense also exist in the plant–oomycete interactions. The specific cell death in potato guard cells and locked stomatal closure during the *P. infestans* infection process indicate that stomata are indeed involved in the potato defense response to *P. infestans* (Figure 2). The findings that stomata close shortly after *P. infestans* inoculation and that this closure significantly affects the ability of the pathogen to colonize and grow in potato also support the phenomenon of stomata-regulated immunity response in *P. infestans*–potato interaction (Wang et al., 2020). However, our finding that stomata open in response to the presence of *P. infestans* and/or AF from disease plants confirms that the pathogen may in some way be able to overcome or suppress the stomata-mediated defense systems.

Lipids and starch are among the main compounds involved in plant stomatal movement. Their metabolisms in plant guard cells are regulated by environmental conditions, such as light, as seen in our study as well as the reported literatures (Horrer et al., 2016; McLachlan et al., 2016). Under light conditions, TAG is catalyzed to provide ATP for the stomatal opening process, such as the activation of a plasma membrane H^+^-ATPase (McLachlan et al., 2016), while starch degradation changes cell turgor to trigger stomatal opening (Santelia and Lawson, 2016). Similar to those in other plants, potato guard cells contain a large amount of starch and TAG, and apparently, *P. infestans* deploys the same mechanisms to regulate potato stomatal movement by altering metabolic activities of lipids and starch as supported by the reverse relations between the size of stomatal apertures and the abundance of TAG and starch that we observed in the *P. infestans*-infected guard cells. This theory is further supported by the observed associations of stomatal opening with TAG breakdown after the infiltration of healthy potato plants with AF from disease plants, the impaired stomatal aperture size by co-infiltration of the AF with DMP, and the increase of stomatal aperture size by malate supplement. Although this appears to be the likely pathway that is affected by the presence of either *P. infestans* or molecules secreted by this pathogen, the exact mechanism by which *P. infestans* regulates this process is not yet known.

It appears that lipid breakdown and starch degradation are involved in the *P. infestans-*induced stomatal opening cascade at different time points. TAG breakdown was found starting from 8 hpi, which was parallel to the starting time of stomatal opening in potato leaves. While degradation of starch in potato guard cells was first observed at 12 hpi and exhausted by 48 hpi, stomatal apertures reached their maximum diameter and failed to respond to stimulation by ABA, SNP, CaCl_2_, Na_3_VO_4_, and H_2_O_2_. It is likely that the energy generated by TAG catabolism (McLachlan et al., 2016) activates a proton-pump H^+^-ATPase and initiates stomatal opening processes, while the subsequent starch degradation reinforces the opening process by strengthening guard cell turgor to maximize stomatal opening (De Angeli et al., 2013).

H_2_O_2_ and NO are important signaling molecules in regulating plant defense responses. Their production in many parts of plants can be triggered upon the recognition of pathogens (Mehdy, 1994; Bolwell, 1999). However, we observed that H_2_O_2_ and NO concentrations in guard cells were sustainably reduced after *P. infestans* infection and AF infiltration, indicating that the metabolism of these molecules in guard cells is independent from their metabolism in other parts of potato plants, such as mesophyll cells. We noticed that H_2_O_2_ and NO reduction in the *P. infestans-*infected guard cells occurred slightly earlier than TAG breakdown, suggesting that the lipid catabolism might be induced by H_2_O_2_ and NO scavenging. Although we did not have statistical support for the relationship in this study, lipid metabolism induced by oxidative radicals has been documented recently in several species (Xie and Roy, 2012; Jin et al., 2018; Becerril et al., 2019). Further study is needed to confirm this hypothesis.

During plant–pathogen interactions, successful pathogens secrete a range of effectors that act inside (cytoplasmic effectors) or outside (apoplastic effectors) plant cells to suppress or manipulate host defense systems (Haas et al., 2009; Giraldo and Valent, 2013) and promote infection. Forced stomatal opening after the infiltration of AF from disease plants into healthy potato leaves suggests that the pathogen-induced stomatal movement is likely mediated chemically either by molecules released from the pathogen, or by molecules produced in the plant in response to pathogen-derived signals. The AF we applied was extracted from *P. infestans-*infected potato leaves. In addition to ions, metabolites, and proteins of potato, this AF may also contain an array of apoplastic effectors secreted by *P. infestans* and we hypothesized that these apoplastic effectors may turn on the stomatal opening pathway through H_2_O_2_ and NO scavenging and TAG breakdown in potato. Cytoplasmic effectors, or other as yet unknown molecules may also directly or indirectly participate in the *P. infestans*-induced stomatal opening pathway since our experiments revealed that the AF alone did not induce starch degradation or stomatal opening to the same degree as that seen during infection (Figure 9).

Since stomatal opening appears to be important for the production of sporangia (secondary inoculum) and not race specific (Supplementary Figure 3), we hypothesized that conserved effectors that are essential for oomycete virulence may have a role in directing stomatal opening. PITG_11755 (protein ID D0NIG7) has been demonstrated to be secreted from *P. infestans* haustoria and has a hypothesized function in the apoplast (Meijer et al., 2014; Wang et al., 2018). *Phytophthora* suppressor of silencing 2, PSR2, encoded by PITG_15152 in *P. infestans* is one of only four effectors so far identified as conserved across members of the *Phytophthora* Genus (Win et al., 2007), suggesting that this cytoplasmic RXLR effector may have an essential role in oomycete pathogenicity. PSR2 and the related *P. infestans* gene PITG_14054, have been shown to function as suppressors of host gene silencing in *P. infestans*–host interactions (de Vries et al., 2017,Vetukuri et al., 2017). The increase of stomatal aperture after PITG_11755 and PITG_15152 overexpression suggests that both of them might participate in stomatal opening during the complex antagonistic interactions between potato guard cells and *P. infestans*. However, further experiments are required to confirm this hypothesis.

Based on these observations, we conclude that potato mounts a defense response against *P. infestans* infection by closing stomata and that *P. infestans* has evolved mechanisms to overcome this defense response. We propose that a series of chemical cascades are involved in the stomatal opening pathway induced by *P. infestans* (Supplementary Figure 8). It starts from the released effector proteins, or other molecules from *P. infestans* that inhibit H_2_O_2_ and NO biosynthesis or promote their catabolism. The lipid breakdown that follows then generates ATP for stomatal opening processes, including through the activation of a plasma membrane H^+^-ATPase. Subsequent starch degradation and glucose and malate accumulation reinforce the stomatal opening to maximum, which later allow the pathogenic sporangiophores to exit the plant and disperse sporangia for the subsequent spread of the disease.

From the perspective of disease epidemiology, quick discharge of enough sporangiophores is essential for rapid spread of potato late blight in a field. In this study, we found that the antagonistic interactions between potato guard cells and *P. infestans* started at a very early time point of the infection course. The closure of potato stomata caused by guard cell suicide in several potato varieties with different resistance levels suggests that potato guard cells can actively respond to the *P. infestans* infection and prevent the releasing of sporangiophores from stomata. However, *P. infestans* can bypass this impediment by manipulating diverse cell processes (directly or indirectly) to open other stomata to maximize apertures for sporangiophore release. The underlying mechanisms of this zig-zag of interactions between potato guard cells and *P. infestans* are likely to be complex and may involve several effectors. Although its detailed mechanisms are not clear yet, we have shown that the stomatal opening is an important pathogenicity strategy for *P. infestans*. Manipulation of stomatal immunity may be an important strategy for future control of potato late blight without agrochemical inputs. More research should be focused on this pathogenicity process to uncover the specific underlying mechanisms that *P. infestans* uses and how they might be disrupted to sustainably control late blight disease.

## Data Availability Statement

The original contributions presented in the study are included in the article/Supplementary Material. Further inquiries can be directed to the corresponding author/s.

## Author Contributions

JZ, L-NY, and ZW: conceived and designed the experiments. L-NY, HL, Y-PW, and JS: performed the experiments. L-NY, JZ, and LG-B: analyzed data and wrote and critically revised the manuscript. All authors reviewed the manuscript.

## Conflict of Interest

The authors declare that the research was conducted in the absence of any commercial or financial relationships that could be construed as a potential conflict of interest.
