# Supplementary material for: Pathogen-Mediated Stomatal Opening: A Previously Overlooked Pathogenicity Strategy in the Oomycete Pathogen Phytophthora infestans
Source: Front Plant Sci. 2021 Jul 12;12:668797. doi: 10.3389/fpls.2021.668797 (PMC8311186; doi:10.3389/fpls.2021.668797)
Supplement: Supplementary file 1 [file Image_1.pdf]

Supplementary Material

Supplementary Figures

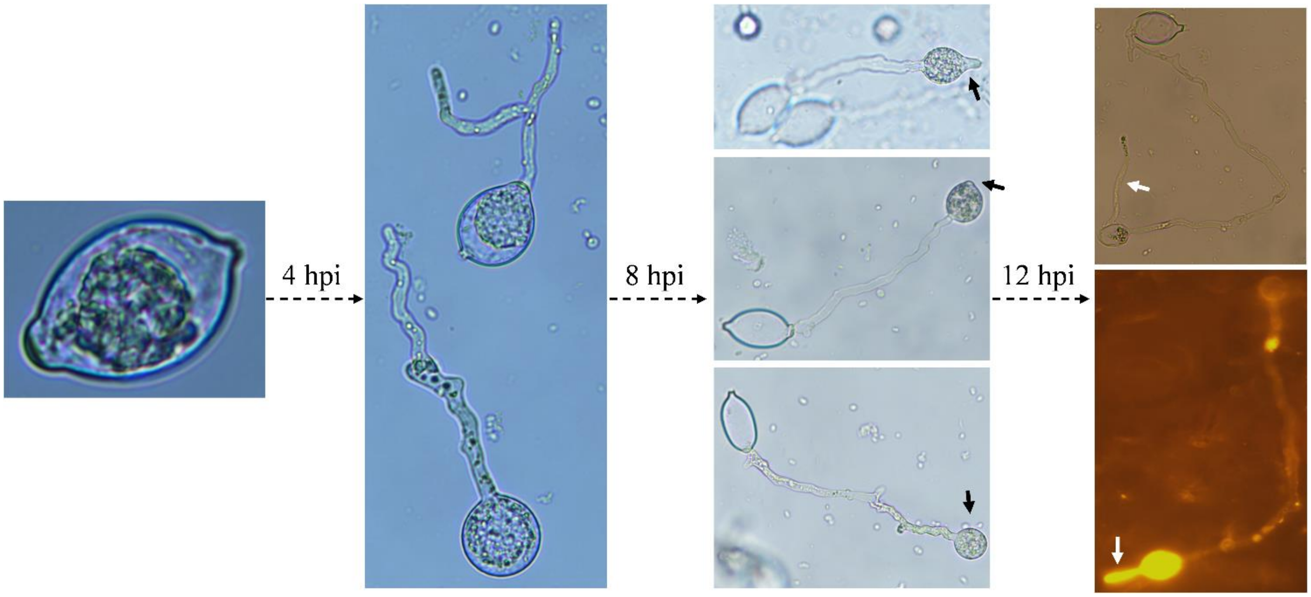

Supplementary Figure 1 Under ideal conditions, *P. infestans* sporangia will germinate within 4 hours, the appressorium (highlighted by black arrows) can be formed within 8 hours and the invading hypha (highlighted by white arrows) can be formed within 12 hours.
