# Supplementary material for: Pathogen-Mediated Stomatal Opening: A Previously Overlooked Pathogenicity Strategy in the Oomycete Pathogen Phytophthora infestans
Source: Front Plant Sci. 2021 Jul 12;12:668797. doi: 10.3389/fpls.2021.668797 (PMC8311186; doi:10.3389/fpls.2021.668797)
Supplement: Supplementary file 2 [file Image_2.pdf]

Supplementary Material

Supplementary Figures

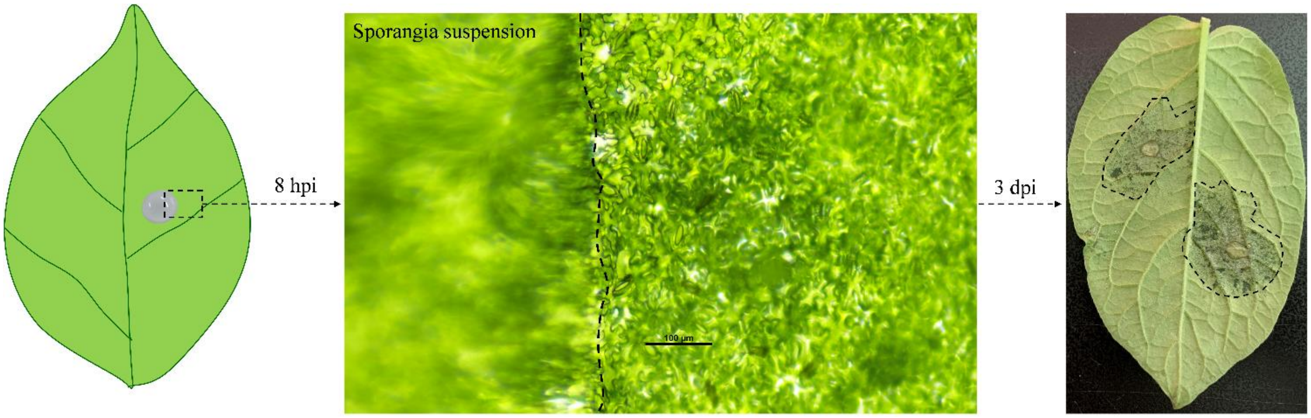

Supplementary Figure 2 After *P. infestans* sporangia suspension (20 µL droplet of 40,000 sporangia per mL) inoculation, stomata started to open radially with the center of sporangia suspension droplet 8 hpi, sporangiophores and sporangia can be found on the surface of the foliage 3 dpi under ideal conditions.
