# Supplementary material for: Pathogen-Mediated Stomatal Opening: A Previously Overlooked Pathogenicity Strategy in the Oomycete Pathogen Phytophthora infestans
Source: Front Plant Sci. 2021 Jul 12;12:668797. doi: 10.3389/fpls.2021.668797 (PMC8311186; doi:10.3389/fpls.2021.668797)
Supplement: Supplementary file 3 [file Image_3.pdf]

Supplementary Material

Supplementary Figures

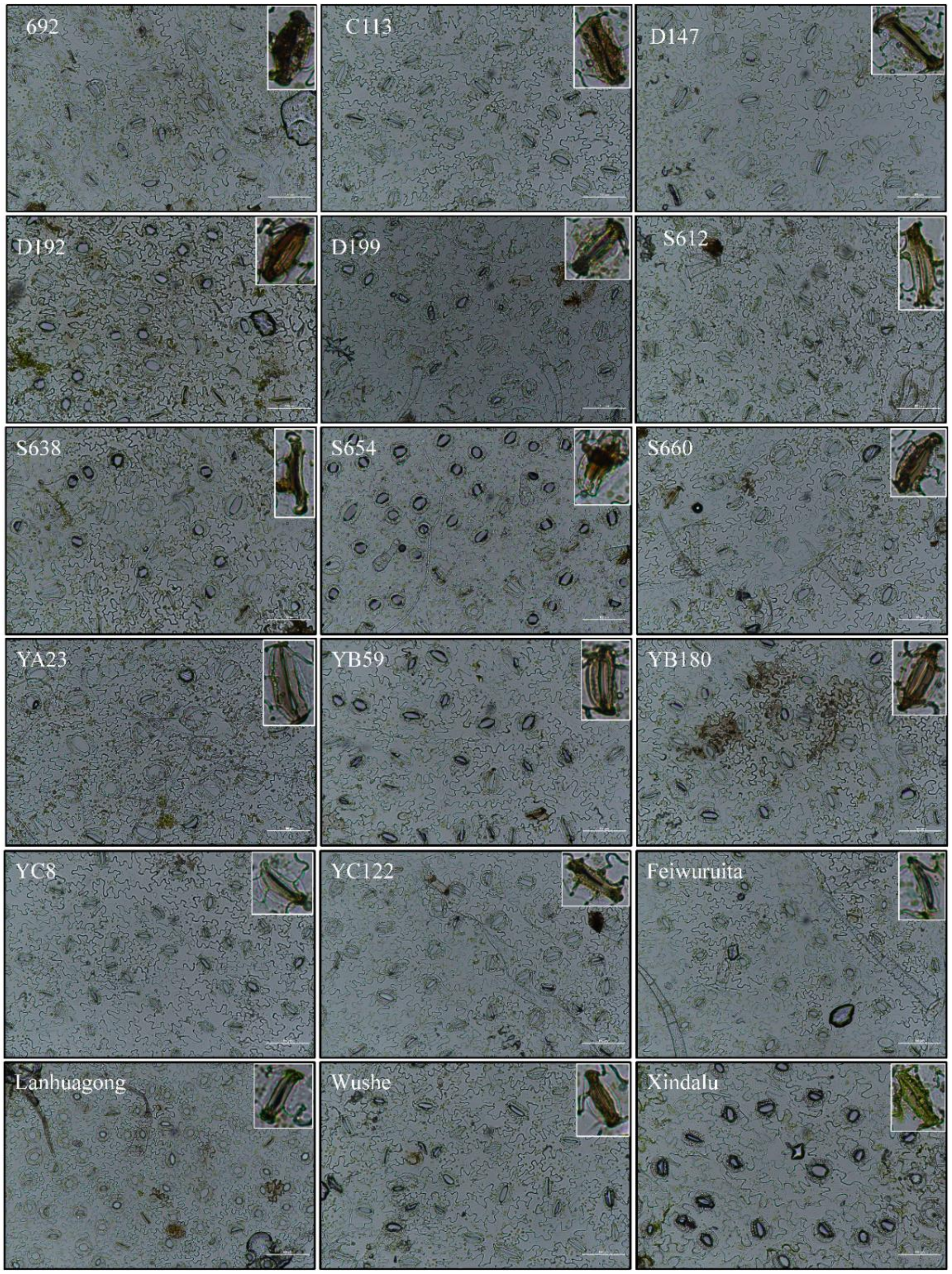

Supplementary Figure 3 *P. infestans* infection induced stomata opening and the hypersensitive guard cell death were found in eighteen different potato cultivars with varying resistance levels. The marker = 100 μm.
