# Supplementary material for: Pathogen-Mediated Stomatal Opening: A Previously Overlooked Pathogenicity Strategy in the Oomycete Pathogen Phytophthora infestans
Source: Front Plant Sci. 2021 Jul 12;12:668797. doi: 10.3389/fpls.2021.668797 (PMC8311186; doi:10.3389/fpls.2021.668797)
Supplement: Supplementary file 4 [file Image_4.pdf]

Supplementary Material

Supplementary Figures

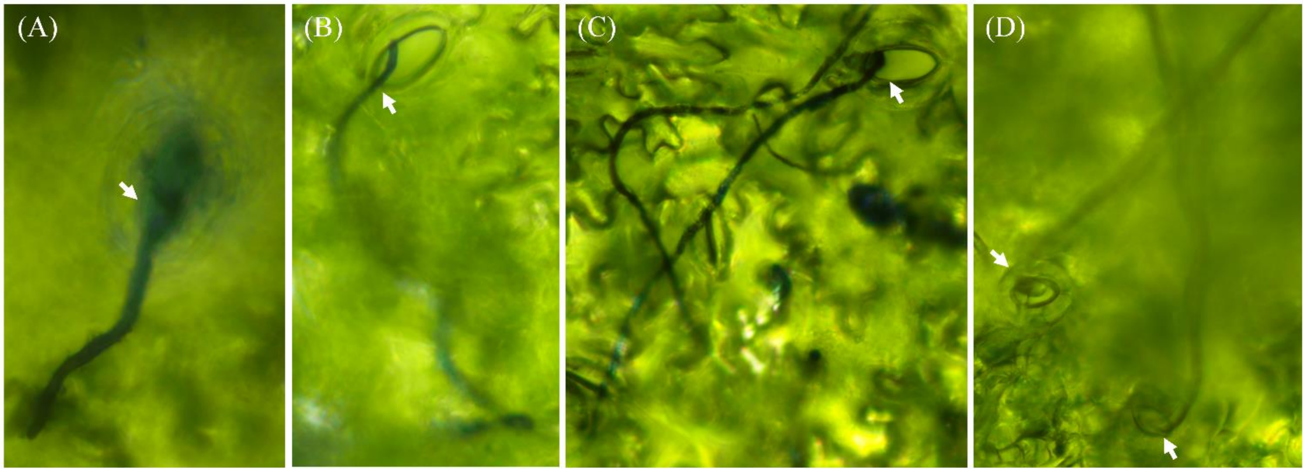

Supplementary Figure 4 Images showing sporangiophores emerged from open potato stomata at 4-5 days post inoculation.
