# Supplementary material for: Pathogen-Mediated Stomatal Opening: A Previously Overlooked Pathogenicity Strategy in the Oomycete Pathogen Phytophthora infestans
Source: Front Plant Sci. 2021 Jul 12;12:668797. doi: 10.3389/fpls.2021.668797 (PMC8311186; doi:10.3389/fpls.2021.668797)
Supplement: Supplementary file 5 [file Image_5.pdf]

Supplementary Material

Supplementary Figures

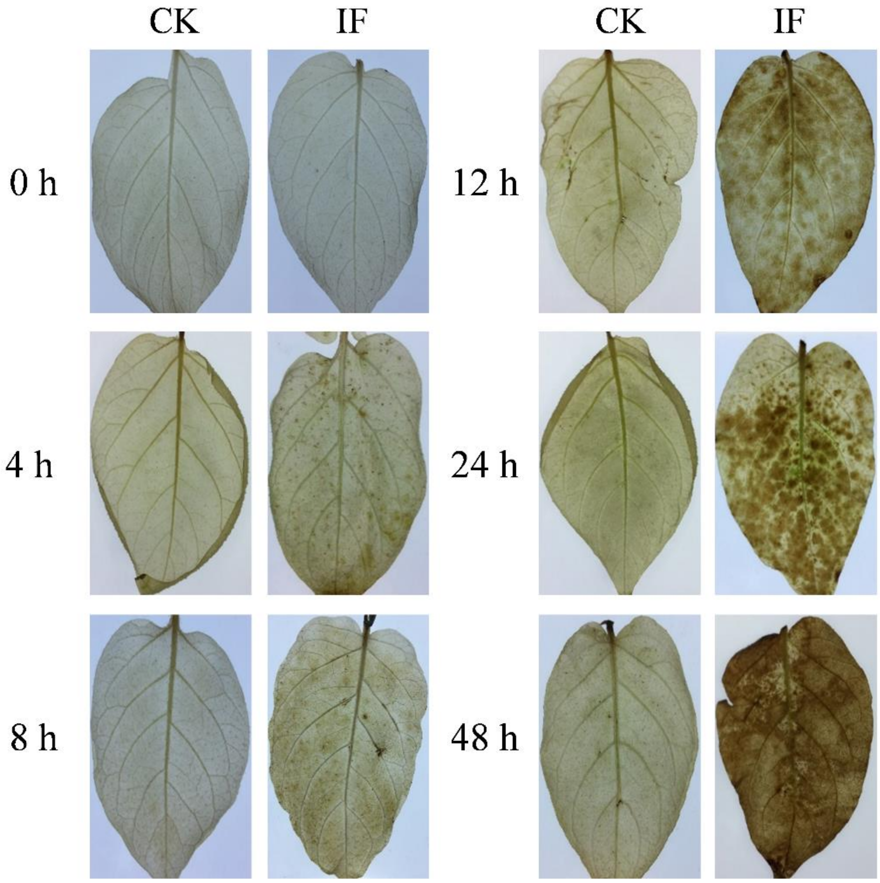

Supplementary Figure 5 The accumulation of H_2O_2 significantly increased in mesophyll cells following *P. infestans* infections.
