# Supplementary material for: Pathogen-Mediated Stomatal Opening: A Previously Overlooked Pathogenicity Strategy in the Oomycete Pathogen Phytophthora infestans
Source: Front Plant Sci. 2021 Jul 12;12:668797. doi: 10.3389/fpls.2021.668797 (PMC8311186; doi:10.3389/fpls.2021.668797)
Supplement: Supplementary file 6 [file Image_6.pdf]

Supplementary Material

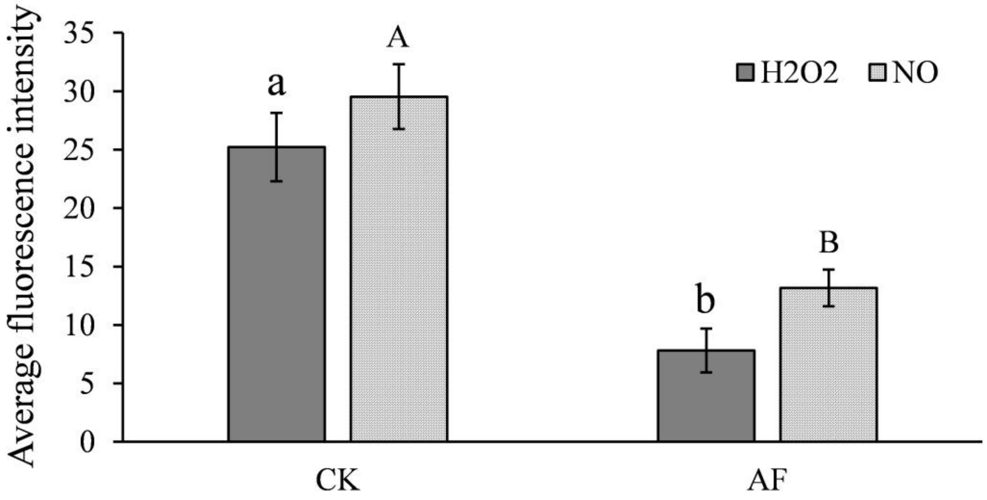

Supplementary Figure 6 Apoplastic fluid (AF) treatment significantly reduced the concentration of H_2O_2 and NO in potato guard cells in 4 of the 6 replicated. In other two replications, AF treatment only slightly reduced H_2O_2 and NO.
