# Supplementary material for: Pathogen-Mediated Stomatal Opening: A Previously Overlooked Pathogenicity Strategy in the Oomycete Pathogen Phytophthora infestans
Source: Front Plant Sci. 2021 Jul 12;12:668797. doi: 10.3389/fpls.2021.668797 (PMC8311186; doi:10.3389/fpls.2021.668797)
Supplement: Supplementary file 7 [file Image_7.pdf]

Supplementary Material

Supplementary Figures

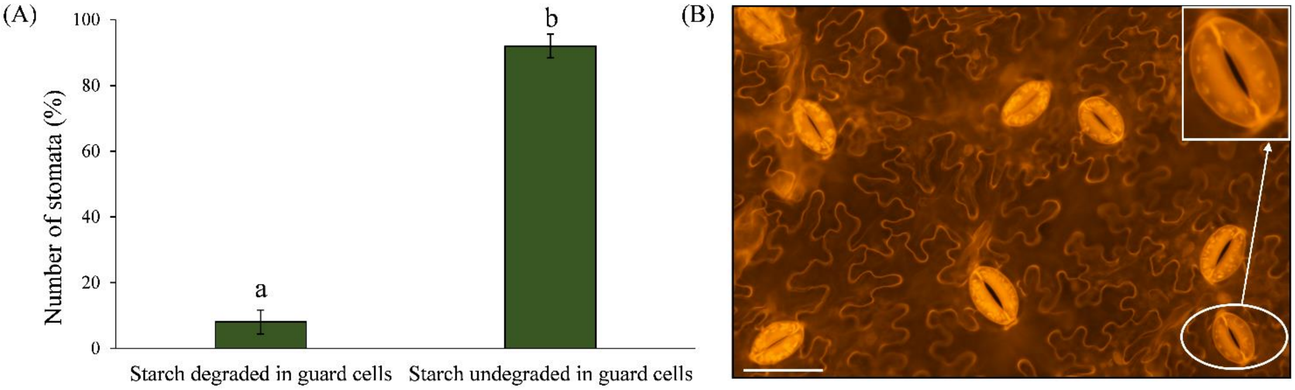

Supplementary Figure 7 Starch reduced in small amount of guard cells after AF infiltration. (A) The percentage of stomata with starch degraded/undegraded in guard cells; (B) image shows starch only degraded in partial of the guard cells (the one in the circle) after AF treatment. The marker = 100 µm.
