# Supplementary material for: Pathogen-Mediated Stomatal Opening: A Previously Overlooked Pathogenicity Strategy in the Oomycete Pathogen Phytophthora infestans
Source: Front Plant Sci. 2021 Jul 12;12:668797. doi: 10.3389/fpls.2021.668797 (PMC8311186; doi:10.3389/fpls.2021.668797)
Supplement: Supplementary file 8 [file Image_8.pdf]

Supplementary Material

Supplementary Figures

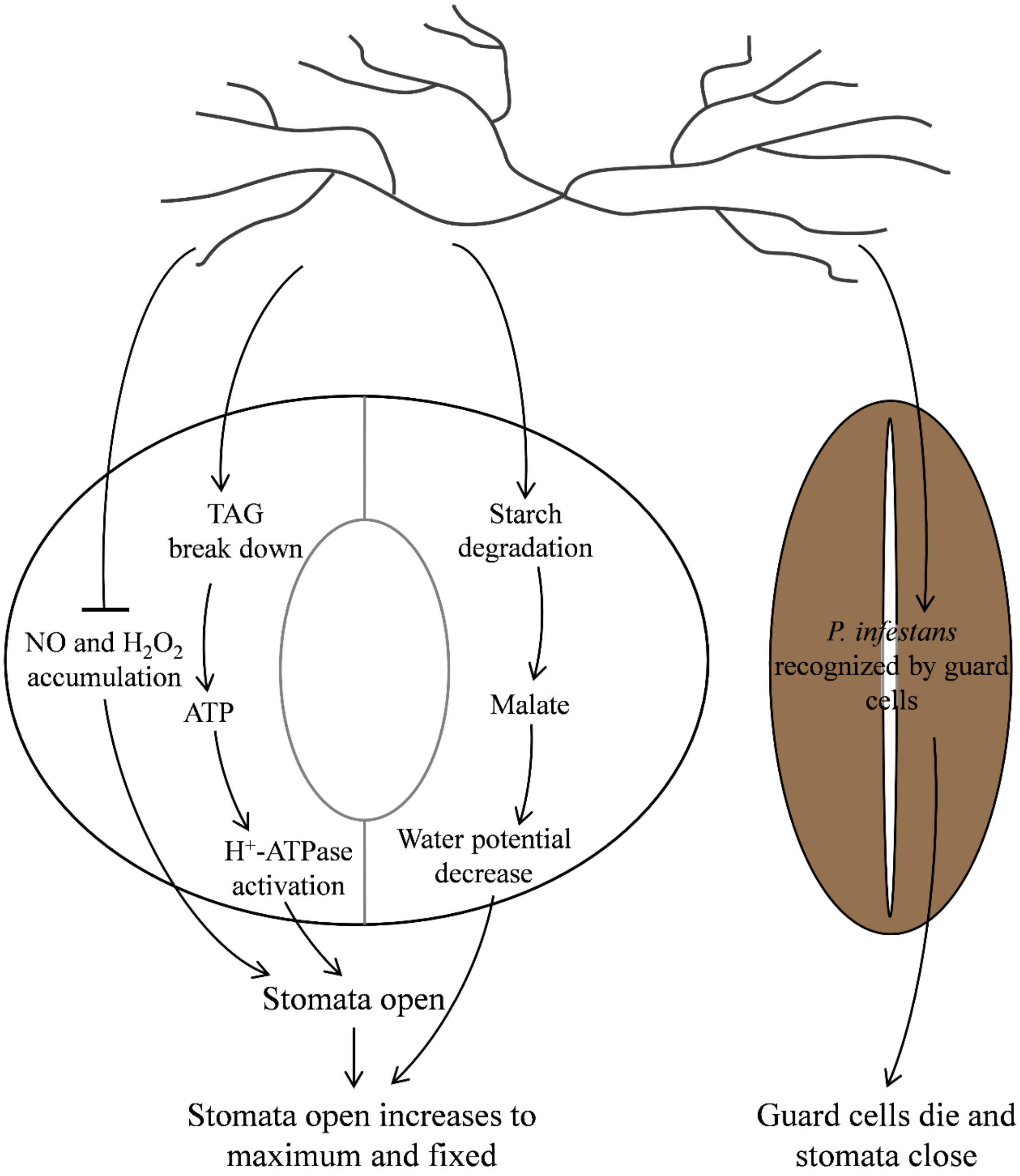

Supplementary Figure 8 Proposed model for *P. infestans* induced stomatal defense and opening. When *P. infestans* sporangia lands on potato leaves, the guard cells can sense the pathogen and close stomata. To overcome the stomatal defense, H_2O_2 and NO biosynthesis was inhibited or the catabolism of them was promoted, induce lipid breakdown to generate ATP for activation of proton pump such as plasma membrane H^+-ATPase required for stomatal opening. Subsequent starch catabolic pathway activated reinforces the stomatal opening by producing malate to freeze the opening.
